# Differences in Art Appreciation in Autism: A Measure of Reduced Intuitive Processing

**DOI:** 10.1007/s10803-022-05733-6

**Published:** 2022-09-05

**Authors:** Mark Brosnan, Chris Ashwin

**Affiliations:** Department of Psychology, Centre for Applied Autism Research, University of Bath, Bath, BA2 7AY UK

**Keywords:** Autism, Intuition, Deliberation, Dual process theory, Art appreciation

## Abstract

Art appreciation reflects an initial emotional and intuitive response to artwork evaluation, although this intuitive evaluation can be attenuated by subsequent deliberation. The Dual Process Theory of Autism proposes that individuals with Autism Spectrum Disorder (ASD) have a greater propensity to deliberate and reduced intuition compared to matched controls. Evaluations of high- and low-quality artworks were undertaken by 107 individuals with a diagnosis of ASD and 145 controls. Controls consistently evaluated high-quality artworks to be much better quality than the low-quality artworks, reflecting intuitive processing. The ASD sample showed a reduced difference in evaluations between high- versus low-quality artwork, which reflects reduced intuitive processing and greater deliberative processing and is consistent with predictions by the Dual Process Theory of Autism.

Art appreciation has been defined as an aesthetic experience which incorporates emotional elements (Funch, 1997). Key theories of art appreciation propose two-factor models that involve an interaction of cognitive and emotional factors that, together, influence the aesthetic appreciation towards evaluation of what represents good versus bad artwork (Chatterjee, 2003; Leder et al., 2004). A theory by Leder et al. (2004) proposes that during early stages of aesthetic perception, viewers quickly and automatically assess the perceptual features of the art. This initial stage is followed by more explicit processing of stimulus features, which includes evaluation of the artwork's content, style and artistic execution. At this latter explicit stage, evaluations are established by viewers' thoughts about the work, incorporated with their emotional responses to it produced by the initial implicit processing stage (Leder et al., 2004, 2014; Scherer, 2005). Another two-factor model of art appreciation (Lindell & Mueller, 2011) proposes a distinction between more bottom–up versus more top–down factors used towards the evaluation of art. Bottom-up factors involves the perceptual processing of the artwork, examining aspects such as the style, symmetry, form, etc. Top–down cognitive factors involve individual differences such as peoples’ art expertise, along with other aspects of the context such as the artwork’s novelty.

Consistent across these two-factor models, art viewers typically rely more heavily on the initial factor. Initial, implicit, bottom-up processing informs the evaluation of high- and low-quality artworks (Leder et al., 2012; Leder et al., 2004, 2014). The automatic nature of this evaluation is reflected in it being characterised as an intrinsic gut instinct response when evaluating works of art (Leder et al., 2014), highlighting the importance of emotional response for aesthetic experience of artwork as fundamental (Leder et al., 2012; Schabmann et al., 2016). Subsequent deliberation appraising stylistic, formal and contextual (e.g., art historical context) aspects (Cupchik & Laszlo, 1992; Leder et al., 2004; Scherer, 2005) attenuates the immediate impact of initial emotions in art appreciation (Leder et al., 2004, 2012; Silvia, 2013). This detaching from the emotional impact of the artwork reflects the later stage, explicit, top–down factor of the two-stage models and only becomes significant when there is sufficient time to engage these subsequent processes in the evaluation of high- and low-quality art (Leder et al., 2006; see also Belke et al., 2006).

The two-stage theories of art appreciation explained above are conceptually similar to more general two-stage theories of human cognition, such as Dual Process Theories. Dual Process Theory has been a dominant model within cognitive psychology for over 50 years (Evans & Frankish, 2009). The dual processes are referred to as Type 1 and Type 2 and can be referred to as intuition and deliberation (respectively). Intuition involves rapid, effortless, parallel, non-conscious, implicit processing that is independent of working memory and cognitive ability. Deliberation, on the other hand, involves slower, effortful, sequential, conscious, explicit processing and is heavily dependent on working memory and related to individual differences in cognitive ability (see Evans, 2011, 2019; Evans & Stanovich, 2013; Kahneman, 2011; Stanovich & West, 2000, 2008; for reviews; see Keren & Schul, 2009 for critique; see Kruglanski & Gigerenzer, 2011 for an alternative view). Within Dual Process Theory, rapid autonomous processes (‘intuition’) are assumed to yield default responses, unless they are intervened upon by distinctive higher order reasoning processes (‘deliberation’). The default-interventionist position is the idea that intuitive processes occur first and precede deliberative processing (see Evans & Stanovich, 2013; Kahneman, 2011).

Dual Process Theory has been applied to the study of the psychology of art appreciation. Dijkstra et al. (2012) developed a paradigm to explore the relative impact of intuitive and deliberative processing on art appreciation, specifically preferences for high-quality over low-quality modern paintings. Participants were asked to make quality judgements varying on a 100-pont scale from *very bad* to *very good* about eight paintings, four of which were high-quality and four being low-quality, as judged by a panel of art experts. Participants were either in an intuitive condition where they had to answer immediately about their evaluation of the paintings, or were in a deliberative condition where they had to deliberate for 1 min about each painting. Dijsktra et al. found that participants in the intuitive condition were better at differentiating between high- and low-quality art than participants in the deliberative condition. Dijskstra et al. replicated this finding with judgements of high- and low-quality poems, concluding that relying on intuition in judgments and decisions about art distinguishes high-quality from low-quality art. In contrast, utilising a deliberative style can serve to hinder the evaluation of quality, yielding more logically consistent responses in many judgement and decision machining situations. Deliberating just before making evaluations about high- versus low-quality paintings is shown to affect the ability to make consistent preferences during these judgements (Nordgren & Dijksterhuis, 2009). A failure to distinguish between high- and low- quality art in these instances reflects the attenuating effect of deliberation on art appreciation (Dijkstra et al., 2013; Nordgren & Dijksterhuis, 2009; Wilson & Schooler, 1991).

Thus, it has been proposed that evaluating high- and low-quality artwork is an emotional, intuitive process, which is often undertaken without deliberation by lay viewers (Dijkstra et al., 2012; Leder et al., 2004). Within the art evaluation paradigm described above (Dijkstra et al., 2012), greater discrimination in the evaluation of high- and low-quality art involves greater emotional response to the art which reflects rapid intuitive processing. In contrast, a reduction in discrimination between the evaluations of high- and low-quality art reflects an attenuation of this initial intuitive response through involvement of deliberative processing. In this way, evaluating high-quality art over low-quality art can be seen as an illustration of the default-interventionist position within Dual Process Theories (see Evans & Stanovich, 2013; Kahneman, 2011).

The Dual Process Theory of Autism proposes relative strengths in deliberative reasoning and decision making and reduced intuitive processing in individuals with a diagnosis of Autism Spectrum Disorder (Ashwin & Brosnan, 2019; Brosnan et al., 2016, 2017; Lewton et al., 2019). Autism Spectrum Disorder (ASD) is characterised by difference in social communication and interaction combined with a pattern of restricted and repetitive behaviours, interests and activities (APA, 2013; WHO, 2018). Evidence for the Dual Process Theory of Autism has been shown through a more ‘logically consistent’ style of thinking by individuals with a diagnosis of ASD than control groups without ASD (De Martino et al., 2008), also characterised as ‘circumspect reasoning bias’ (Brosnan et al., 2014). Individuals with a diagnosis of ASD also show less susceptibility to the biases which are associated with intuitive processing (Farmer et al., 2017; Fujino et al., 2019; Shah et al., 2016; see Kahneman, 2011). Dual process Theory has been applied to autism to propose that challenges with social communication and interaction arise from difficulties engaging with intuitive processes that are typically involved in many facets of social interaction (Ashwin & Brosnan, 2019; see also Rand et al., 2014).

The Dual Process Theory of Autism would predict that the style shown by individuals with a diagnosis of ASD characterised by enhanced deliberation and reduced intuition would lead to reduced differentiation in evaluating between high- and low-quality art in the paradigm developed by Dijkstra et al. (2012). We hypothesised that control participants, however, would show the typical discrimination between the low- and high-quality art, more specifically, that the control sample would utilise intuition to provide better evaluations for high-quality art over low-quality art.

## Methods

### Participants

All the participants were studying for A-level examinations (taken at age 18, used as entry requirements for university) within the UK. The study recruited 107 participants with a diagnosis of ASD (71 male, 31 female, 5 non-binary/trans; mean age = 17.8 years; sd = 2.1) from attendees at a summer school for young autistic people transitioning to University, and 145 control participants [88 male, 57 female; mean age = 17.4 years (sd = 1.9)] who had no self-reported neurodevelopmental disorders and were recruited from a general summer school for university transition. Participants were recruited from five different summer schools for each sample, with approximately 20–30 participants recruited from each summer school. Participants with a diagnosis of ASD had to provide documentation relating to their diagnosis in order to attend the autism summer schools, which was checked and verified by the administration team. Although this data was not available for research purposes, the documentation confirmed that a formal diagnosis of ASD had been provided by teams of clinicians following DSM or ICD criteria (APA, 2013; WHO, 2018). The proportion of males to females did not significantly differ between the two groups (Chi^2^(1) = 2.08, ns), and no significant difference between the two groups was found for age [t(240) = 1.31, ns].

### Measures

The methods in the present study followed those developed by Dijkstra et al. (2012), including the images of paintings used as stimuli. A set of eight paintings were used for the task, with four high-quality paintings taken from MoMA (Museum of Modern Art, New York, website: www.moma.org) and four low-quality paintings from MoBA (Museum of Bad Art, Boston, website: www.museumofbadart.org). The eight examples were all paintings of human forms. This division in quality of MoMA and MoBA paintings was confirmed by ratings from three experts in modern art (teachers at the Academy of Art; Cohen's Kappa = 1; see Dijkstra et al., 2012). Paintings were presented individually on a computer in a random order and participants judged the quality of the paintings by rating each on a scale ranging from 0 (very bad) to 100 (very good), Participants were told to rely on their initial judgement and to not think about the judgements too much, but there were no time constraints or recordings of timings (see Dijkstra et al., 2012).

An assessment of autistic traits was undertaken to identify if the levels of autistic traits were consistent with diagnostic status, as groups with a diagnosis of ASD score higher in autistic traits than groups not on the autism spectrum (Ruzich et al., 2015). All participants competed the Sub Autistic Traits Questionnaire (SATQ; Kanne et al., 2012) as a measure of the degree of autistic traits. The SATQ is a 24-item self-report questionnaire that assesses a broad range of subthreshold autism traits that are relevant to the general population and differentiate those on the autism spectrum. The SATQ has a Cronbach's alpha coefficient = 0.73, and a test–retest reliability = 0.79.

The testing session was conducted on a desktop computer using the Qualtrics platform (Qualtrics, Provo, UT) to present the task and questionnaires and record responses. All the summer schools were run by the authors and all the data was collected in the same computer laboratory. The assessments took around 15–20 min to complete. Due to a technical error, the SATQ scores were not processed for one of the autism summer school groups, so the number of completed SATQ scores are lower for this group (n = 76). Ethical approval was provided by the Psychology Research Ethics Committee [PREC], and all participants gave informed consent to take part. Participation was voluntary within the summer schools and there were no rewards for taking part. During debriefing, none of the participants in either group reported having seen the images before or considered themselves to be art experts.

### Analysis

The mean art evaluation rating scores formed the DV for the analyses. A mean rating for the four high-quality and the four low-quality paintings was calculated for each participant to create a mean for each of the two conditions of the task, the high- and low-quality paintings. Condition (high- quality/low-quality) was the within participant factor and Group (autism/control) was the between participant IV for the analyses. A repeated measures ANOVA was run to identify if the difference between evaluations of high- and low-quality art varied between autism and control groups. A partial correlation (controlling from group) was then conducted to explore the relationship between autistic traits and the ratings of good and bad art.

## Results

Consistent with diagnostic status, the mean scores on the SATQ showed a significant difference between the autism group (m = 38.75; sd = 10.26) and the control group (m = 24.94 (sd = 9.20), t(219) = 10.18, p < 0.001), showing that the autism group had a much higher degree of autistic traits than the control group, as expected. Both the autism and control groups had comparable SATQ means in the present study to previously published means for control and autism samples (e.g. Kanne et al., 2012).

**Fig. 1:**
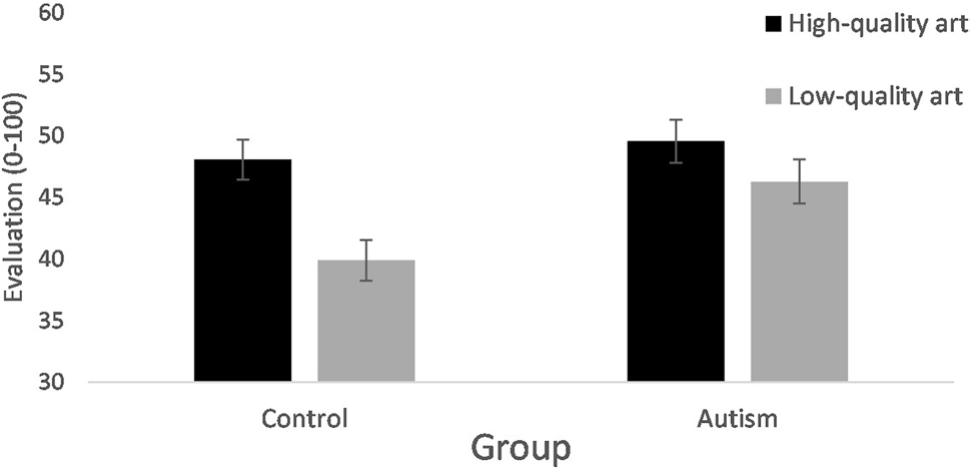
Mean evaluation of high- and low-quality art by autism and control groups

The test of between participant contrasts from the repeated measures ANOVA revealed no significant main effect of Group [F(1,249) = 2.82, ns], indicating that the autism and control groups provided comparable evaluations overall about the art. The ANOVA also revealed for within participant contrasts that there was a significant main effect for Condition; [F(1,249) = 46.09, p < 0.001] showing that the high-quality art had significantly better ratings compared to the low-quality art. In addition, there was a significant interaction for Condition with Group [F(1,249) = 7.80, p = 0.006]. The control group provided significantly better evaluations for high-quality art compared to low-quality art, whereas this discrimination was attenuated in the evaluations of the autism group (see Fig. 1). Post-hoc within samples t-tests highlighted that the control group rated the high-quality art significantly better than the low-quality art [t(143) = 8.22, p < 0.001; Cohen’s d = 0.7] and this effect was attenuated in the autism group [t(106) = 2.34, p = 0.021; Cohen’s d = 0.2]. Consistent with this, post hoc between-group analyses revealed that the autism and control groups did not significantly differ on evaluating high-quality art [t(249) = 0.66, ns], but they did significantly differ on rating low-quality art [t(250) = 2.53, p = 0.012] with the control group making lower evaluations about the low-quality art than the autism group.

Finally, to explore the role of autistic traits and art appreciation in the task, a partial correlation was conducted between autistic traits and the ratings of good and bad art. The partial correlation controlled for group (autism/control) as significant differences were identified between the groups. The degree of autistic traits showed a significant negatively correlation with the ratings for both good art [r(215) = − 0.23, p = 0.001] and bad art [r(215) = − 0.23, p = 0.001].

## Discussion

The ability to distinguish high-quality art versus low-quality art is argued to be an initial, emotional intuitive process, which can then be attenuated by subsequent deliberation (Dijkstra et al., 2012, 2013; Nordgren & Dijksterhuis, 2009; Wilson & Schooler, 1991). Consistent with theories of art appreciation (Leder et al., 2004, 2012; 2014), the present study found that the control group consistently evaluated high-quality art as much better than the low-quality art, an effect which is argued to reflect a reliance upon initial intuitions (or a ‘gut response’) in evaluating artworks. The discrimination between high and low quality art by the control group had medium to large effect size.

The present study also found that the art evaluations by the autism group reflected a reduced distinction between high- and low-quality art compared to the control group, with the discrimination for the autism group having a small effect size compared to a medium to large effect size for the controls. While the differences between high and low-quality art were significant for both groups, the difference between high and low-quality art evaluations was attenuated in the autism group as demonstrated by the different effect sizes. Additionally, the autism group had significantly higher ratings for low-quality art than the control group, reflecting evaluations of low-quality art by the autism group being more similar to their ratings for the high-quality art. In research from the general populations, reduced discrimination like this is argued to reflect the subsequent effect of greater deliberation on the evaluation of the art over intuitive processes (Dijkstra et al., 2012, 2013; Leder et al., 2004, 2012, 2014; Nordgren & Dijksterhuis, 2009; Wilson & Schooler, 1991). Previous research has found that the initial intuitive evaluation of high-quality art over low-quality art is attenuated in non-ASD groups when experimental manipulation encourages deliberation (Dijkstra et al., 2012). In the present study, reduced discrimination of high- and low-quality art by the autism group is consistent with the Dual Process Theory of Autism which proposes a reduction in initial intuitive processing and greater deliberation in autism compared to controls (Ashwin & Brosnan, 2019; Brosnan et al., 2016, 2017; Lewton et al., 2019). This was found despite participants being told to rely on their initial judgements and not to think about their judgements too greatly, which should facilitate intuitive responding. However, this was not the case here for the autism group, who showed effects indicative of over-relying on deliberative over intuitive processing. Using this art appreciation paradigm indicates that the control group defaulted to an evaluation reflective of intuitive processing whereas the autism group defaulted to an evaluation reflective of deliberative processing.

The Dual Process Theory of Autism proposes that the default interventionist position is attenuated or absent in autism, resulting in defaulting to deliberative processing. This results in more logical accurate responses, less influenced by potentially erroneous biases in groups with a diagnosis of ASD (Brosnan et al., 2016, 2017; De Martino et al., 2008; Farmer et al., 2017; Fujino et al., 2019; Lewton et al., 2019; Shah et al., 2016). The pattern of the autism group reflecting an attenuated pattern of the control group effects (see Fig. 1 and a small within-participants effect in the autism group and a medium to large within-participants effect in the control group) is suggestive of an attenuated rather than absent default-interventionist position for individuals with a diagnosis of ASD. Importantly, these findings are not attributable to individuals with a diagnosis of ASD performing differently in general to the control group on the task, such as not understanding the task or having difficulty evaluating art quality (and the group means are similar to those reported for intuitive and deliberative conditions by Dijkstra et al., 2012). Indeed, there was not a significant between-participant effect overall between the groups for the ratings of art in general or the ratings of high-quality art specifically. The difference in the discrepancy of the evaluations of high- and low-quality art was significant for the autism group (p = 0.021) albeit attenuated compared to the effect for the control group (p < 0.001).

Deliberative processing is argued to attenuate, rather than enhance, initial intuitive evaluations due to the complexity of factors that influence judgements and decisions about the quality of art (Dijkstra et al., 2012, 2013; Nordgren & Dijksterhuis, 2009; Wilson & Schooler, 1991). Participants here were all non-art experts and would find challenging evaluation of the artwork's content, style, symmetry, form and artistic execution reflective of the later stages of processing disruptive (Leder et al., 2004, 2014; Lindell & Mueller, 2011; Scherer, 2005). Importantly, within this art appreciation paradigm, the *intuitive* response was consistent with the judgements of art experts (Dijkstra et al., 2012; 2013). Leder et al. (2014) argue that art expertise results in an attenuation of the initial emotional, intuitive response by subsequent explicit processing of stimulus features. Thus, whilst deliberation may consistently inform art appreciation for those with art expertise, deliberation can also be disruptive for non-art experts making complex judgements and decisions about art. It is also important to note the intuitive response of non-art experts matched that of art experts as there are few measures of intuitive responding, and measures of intuition are often inferred from erroneous biases made on reasoning tasks (Pennycook et al., 2016a, 2016b). This art paradigm provides an index of intuitive processing that accords with experts, rather than being errorful.

Shaughnessy (2013) describes a ‘neurodivergent aesthetic’ whereby, for people who feel like ‘outsiders’, the appeal of certain artworks might lie in their capacity to inspire a fantasy of participation. As such, art appreciation is revealed, not as a peripheral supplement to human experience, but as a privileged medium of human contact (c.f. Cardinal, 1994, 2010). The present study did not identify what aspects of the art were appreciated, although the findings of this study do suggest that the ‘neurodivergent aesthetic’ experience of the autism group differs from the aesthetic experience of the control group. This difference was particularly evident with respect to a reduced difference in the evaluation of high-quality versus low quality art by the autism group compared to the control group, which was driven by a relatively less negative evaluation of low-quality art by the autism group. Leder et al. (2014) propose that an emotionally distanced mode of art appreciation can enable positive evaluation of art depicting negative images (such as those depicting despair, for example). Thus, future research can explore whether a neurodivergent aesthetic reflects an emotionally distanced mode of art appreciation (reflective of greater deliberative/reduced intuitive processing). It may also be that art created by autistic artists has a less emotional aesthetic, or that autistics perceive different aspects of art as better quality compared to neurotypicals, such as art being more systematic (see Roth, 2020). However, it is important to note that the images in the present study represented human representations which may enhance a sense of participating in human contact (see above). In addition, human representations are images with high ‘fluency’ (i.e. viewers can see what is being represented rapidly) which may indicate these findings do not extend to ‘low fluency’ abstract art (in which it is harder to identify what is being represented: Belke et al., 2010; Kuchinke, et al., 2009).

The Dual Process Theory of Autism proposes that those with higher levels of autistic traits are associated with greater deliberative/reduced intuitive processing, in both autistic and non-autistic groups. Consistent with this, for the first time, this study identified a significant relationship between autistic traits and art appreciation. When controlling for group (autism/control), the findings here showed that higher levels of autistic traits were associated with lower ratings for both good and bad art. The strength of the correlation with autistic traits was identical for both good and bad art, which may suggest that a neurodivergent aesthetic (see above) is informed by factors beyond autistic traits alone.

There are some limitations of the study that should also be noted. The participants across both groups were largely around 17–18 years of age and were all thinking about going to university, and so likely to be more academically-able with above average levels of intelligence. However, this was true for both groups, so while levels of intelligence were not assessed here due to time constraints, both groups were expected to have comparably high levels of intelligence comparable to those reported by students attending university. Although art expertise was not formally assessed here, discussions after the task revealed that no participant reported having seen these artworks before nor having a particular expertise in art.

Testing was done in group sessions which was not amenable to timing data, so timing to index about intuitive versus deliberative processing was not suitable despite these measures potentially being informative. Since intuitive and deliberative responses are characterised by rapid and slower processing (respectively), this would propose differences in response time data related to cognitive processing. However, the array of additional processes involved in the current computer-based task (e.g. perceptual, motor, task-related etc.) means a much more tightly controlled experimental condition would be required than was possible here for the use of response time data to support the involvement of intuitive versus deliberative processing in the present testing. There are also methodological issues about accurately measuring the independence of separable processes within repeated measures assessments, since response times can be affected by the success of previous responses (Spiliopoulos, 2018). Furthermore, there is a potential circularity if deliberative processing is inferred solely from longer response times and if longer response times are solely taken to infer deliberative processing, and instead response time increases can better be accounted for when participants are presented options that are harder to discriminate e.g. strength-of-preference or discriminability between choice options (Krajbich et al., 2015). Pennycook et al. (2016b) argue that the detection of conflict with an initial intuitive response is what produces deliberative processing and consequent increases in response time. Evans & Stanovich (2013) have claimed that speed and accuracy are correlated but not central factors in what determines the distinction between intuitive and deliberative processing. The interferences about dual processing that can be drawn from response times are therefore open to debate (see De Boeck & Jeon, 2019, for an overview), but are an interesting factor to investigate in future tightly controlled lab based studies in this area.

Participants in the present study were told to rely on their initial judgement and to not think much about the judgements, in line with previous methods for the task (Dijkstra et al., 2012). We would hypothesise that instructions to deliberate for 1 min before making a judgement would impact upon the discrimination of high-quality art over low quality art in the control group and have no impact upon the autism group. If the default-interventionist position is attenuated rather than absent (as suggested above), rapid presentation of the artworks and a requirement to respond rapidly may enhance the evaluations of high-quality over low-quality artworks by individuals with a diagnosis of ASD. These ideas remain to be tested in further research. Finally, whilst the diagnoses of ASD were confirmed for this group of participants, we did not have access to data that would enable us to explore differences within the autism group (e.g. ADOS scores).

In conclusion, consistent with previous research, the control group distinguished between high- and low-quality art. This is argued to reflect an initial emotional, response to the art. Subsequent deliberation is argued to attenuate this initial response and the autism group did not distinguish to the same degree between high- and low-quality art. These findings are consistent with the Dual Process Theory of Autism which proposes that controls default to intuitive processing and individuals with a diagnosis of ASD default to deliberative processing.
